# Serological Evidence of MERS-CoV Antibodies in Dromedary Camels (*Camelus dromedaries*) in Laikipia County, Kenya

**DOI:** 10.1371/journal.pone.0140125

**Published:** 2015-10-16

**Authors:** Sharon L. Deem, Eric M. Fèvre, Margaret Kinnaird, A. Springer Browne, Dishon Muloi, Gert-Jan Godeke, Marion Koopmans, Chantal B. Reusken

**Affiliations:** 1 Saint Louis Zoo Institute for Conservation Medicine, Saint Louis, Missouri, United States of America; 2 Institute of Infection and Global Health, University of Liverpool, Leahurst Campus, Neston, United Kingdom; 3 International Livestock Research Institute, Nairobi, Kenya; 4 Mpala Research Centre, Nanyuki, Kenya; 5 Wildlife Conservation Society, Global Conservation Programs, Bronx, New York, United States of America; 6 Molecular Epidemiology and Public Health Laboratory, Hopkirk Research Institute, Massey University, Palmerston North, New Zealand; 7 Netherlands Centre for Infectious Disease Control, National Institute for Public Health and the Environment, Bilthoven, the Netherlands; 8 Department of Viroscience, Erasmus Medical Centre, Rotterdam, the Netherlands; The Chinese University of Hong Kong, HONG KONG

## Abstract

Middle East respiratory syndrome coronavirus (MERS-CoV) is a recently identified virus causing severe viral respiratory illness in people. Little is known about the reservoir in the Horn of Africa. In Kenya, where no human MERS cases have been reported, our survey of 335 dromedary camels, representing nine herds in Laikipia County, showed a high seroprevalence (46.9%) to MERS-CoV antibodies. Between herd differences were present (14.3%– 82.9%), but was not related to management type or herd isolation. Further research should focus on identifying similarity between MERS-CoV viral isolates in Kenya and clinical isolates from the Middle East and elsewhere.

## Introduction

Middle East respiratory syndrome coronavirus (MERS-CoV) is a newly emerged betacoronavirus that has been associated with the ongoing reporting of a severe respiratory disease in humans in parts of the Middle East [1], [2]. Accumulating evidence points to dromedary camels (*Camelus dromedarius*), with only mild clinical signs of short duration, as an important reservoir of the virus and as responsible for its transmission to humans [3], [4], [5], [6], [7], [8]. Understanding the epidemiology of MERS-CoV in dromedary camel populations is critical in assessing and managing the risk to humans posed by the circulation of the virus.

Kenya has Africa’s third largest dromedary camel population, estimated at 3,091,200 animals; the camel meat and milk industry in Kenya is worth approximately US$ 11,000,000 annually [9]. Corman et al. (2014) investigated MERS-CoV antibody prevalence in archived serum samples collected from camels throughout Kenya, spanning a period from 1992–2013. They found an overall seroprevalence of 29.5% ranging between 0–17.5% in the northern Rift Valley and 53.4%-100% in the Northeastern and eastern regions of the country [10]. Additionally, nomadic herds and those with higher camel densities were found to have a higher seroprevalence [10].

Laikipia County, Kenya supports a low, but increasing camel population due to changes in human demographics and increasing drought conditions for which camels are better adapted than other livestock [11], [12]. From 2001 to 2012, Laikipia County’s camel population increased by 15% and currently is around 5000 individuals, with projections that point to continued increase [13], [14].

Since 2011 we have conducted camel health studies across a variety of land management regimes in Laikipia County. The objective of this study was to investigate the level of exposure to MERS-CoV in dromedary camels under different herd management systems in Laikipia County.

## Materials and Methods

All sampling procedures were approved prior to the study by the Kenyan National Council of Science and Technology for field studies to be conducted with dromedary camels in Laikipia County, Kenya. Approval for the study was obtained from the Kenyan National Council of Science and Technology (NCST; permit number NCST/RRI/12/1/BS011/064) and the Institutional Animal and Care and Use Committee of the Saint Louis Zoo. Oral consent was obtained from the owners of sampled camels.

Laikipia County covers 9,666km^2^ and is characterized by a rainfall gradient of 900 mm/yr at the equator to 400 mm/yr on the northern boundary (S1 Fig). Land use is typically permanent small-holder agriculture in the south and commercial and group ranches in the north [15]. Three habitats characterize Laikipia: 1) *Acacia drepanalobium* woodlands, 2) grass and shrub savannas, and 3) bushlands dominated by *Acacia mellifera*, *A*. *etbaica*, *A*. *brevispica* and *Grewia tenax*. Land use includes commercial livestock ranches, pastoralist communal lands and wildlife conservancies, though most properties are under a mixed management regime [16]. The camel herds in our study were categorized as follows: five at predominantly commercial ranching properties (i.e., camel milk produced for sell), two at mixed commercial/pastoralist properties (i.e., group herds for camel milk production primarily for subsistence use), and two nomadic herds (i.e., camels used for the movement of supplies and people). Sampling took place from June-August 2013.

We sampled 335 camels from nine herds. Herds were selected opportunistically based on ease of access and willingness of owners to participate in the study. While efforts were made to sample a diverse subset of each population, mainly by selecting a range of ages and sexes, a truly random sampling of the herds was not performed due to logistical constraints. Camels were manually restrained by herders and a 4-8ml blood sample was collected from the jugular vein using an 18 g needle. Restraint was accomplished by hobbling one front leg with a rope so the camel could not kick or walk away. No animals were sacrificed for this study.

Blood was stored on ice for transport to the Mpala Research Centre, where it was centrifuged and serum separated and frozen at -20°C. Samples were shipped on dry ice for testing at Erasmus University, Netherlands. All sera were transported in agreement with Dutch import regulations regarding animal disease legislation. Basic demographic and management data relating to each herd and each camel were collected. Herds were categorized by management type (e.g., commercial, commercial/pastoralist, nomadic) and the degree of isolation from any new camels that enter the herd. Isolation categories included: low isolation (6 or more camels enter herd in 1 yr or camels move around consistently with high probability of interacting with other camels); intermediate isolation (3–5 camels enter herd in 1 yr); and high isolation (1–2 camels enter herd in 1 yr). Ages were assigned as: young (≤ 6 months), juvenile (6 months– 2 years), and adult (> 2 years) based on dental wear and herder/owner knowledge.

Serum samples were tested at a 1:20 dilution for presence of IgG antibodies reacting with MERS-CoV (residues 1–747), severe acute respiratory syndrome (SARS)-CoV (residues 1–676) and human coronavirus (HCoV)-OC43 (residues 1–760) spike domain S1 antigens using extensively validated protein-microarray technology [7], [17]. HCoV-OC43 S1 was used as proxy for bovine CoV (BCoV), which is known to circulate commonly in dromedary camels [7].

Effects of age and herd size on MERS-CoV exposure were analyzed using ANOVA and MANOVA with P<0.05 considered significant (SPSS Version 16.0). Chi-square Tests were used to compare management types and herd isolation levels and MERS-CoV exposure with P<0.05 considered significant (NCSS Version 7).

## Results

Overall mean seroprevalence of MERS-CoV antibodies in the sampled population is 46.9% (95% CI 41.4–52.5) with a prevalence of 60.8% (53.6–67.7) in the adult, 21.3% (12.9–31.8) in the juvenile, and 39.3% (27.1–52.7) in the young cohorts (S2 Fig; S1 Table). All nine herds had at least one positive camel, with the lowest mean herd prevalence of 14.3% (95% CI 4.8–30.3%) and the highest of 82.9% (95% CI 66.4–93.4) (S1 Table).

In addition to MERS-CoV antibodies, there was a high level of circulation of BCoV (based on HCoV-OC43 S1 as a proxy) in the camels as has been previously documented in other dromedary camel populations (S2 Fig) [7], [17]. All samples tested negative for severe acute respiratory syndrome SARS-CoV (S1 Fig). Analyses of exposure by age provides evidence of higher levels in older individuals (F_2,23_ = 2.661 p = 0.09); young animals had a significantly lower prevalence compared to adults (Duncan's test, P<0.05), as well as a trend towards higher prevalence rates in smaller herds (F_1,6_ = 4.23; p = 0.085). There was no statistical effect based on herd management type, with prevalence in commercial herds (43.6%; 35.8–49.6), commercial/pastoralist herds (51.9%; 37.6–66.0) and nomadic herds (56.8%; 44.7–68.2) (*X* ^*2*^; P = 0.1). Additionally, there was no statistical difference in prevalence based on herd isolation with high (40%; 28.2–54.6), intermediate (52%; 41.2–60.5), and low (54%; 44.7–68.2) isolation (*X* ^*2*^; P = 0.6).

## Discussion

Our study demonstrates high levels (46.9%) of seroconversion to MERS-CoV in Laikipia County camels. There was no difference in seropositivity levels between herds based on herd management or isolation type, and antibodies were found in all age cohorts. The seroprevalence across ages in combination with herds categorized as having no or little contact with external herds (e.g., high isolation type), suggests that Laikipia camels continue to be exposed to MERS-CoV or a closely related virus. If exposure was dependent on transmission of virus from outside Laikipia, one would expect a lack of seroconversion in the juvenile cohort (i.e., after maternal antibodies wane).

The trend towards a higher seroprevalence in smaller herds was not correlated with herd management or isolation type and differs from a previous study in Kenya in which the authors suggest camels in more mobile, nomadic herds had higher rates of exposure due to increased horizontal contact (S1 Table) [10]. Differences between our study and Corman et al. (2014) may be due to Laikipia camels residing further from international borders, and thus having less contact with camels from countries with known high seroprevalence [18]. Corman et al. (2014) demonstrated a 10% (7.2–13.5) seroprevalence from archived samples from Laikipia camels collected from 1992–2013, with a 2.5% (0.06–13.2) prevalence from the 2013 samples. Although the diagnostic tests differed between our study and the Corman et al. (2014), it is unlikely that this would account for the extreme difference in prevalence. The seroprevalence of 46.9% in the current study is either comparable or lower than other African countries were MERS-CoV exposure has been studied [17], [18].

One limitation to this study is the convenience sampling scheme which may have led to bias associated with owners allowing samples to be collected from more tractable individuals (e.g., older). However, we sampled across a range of ages and sexes in each herd.

Although Laikipia County camel density is low relative to more northern regions of Kenya, our study suggests the population is sufficient to maintain high rates of viral transmission and that camels may be constantly re-infected and serve as long term carriers of the virus [19]. With recent debate on the role of camels in the transmission of MERS-CoV to humans, studies such as ours, which demonstrate high MERS-CoV exposure in camels, provide support for the need of further research on the role of camels in the epidemiology of this emerging zoonotic disease.

Several researchers have proposed that MERS-CoV, and similar viruses, are likely to have been circulating in camel populations across the Middle East and Horn of Africa for many years, but may not have yet acquired zoonotic potential across their range [4], [10]. Therefore, placing virus diversity in a regional context is an important next step. Determining whether MERS-CoV of Laikipia camels is genetically similar to MERS-CoV in other parts of the Horn of Africa, as well as to the known human pathogenic strain in the Middle East, will help to elucidate if the camel population, along with its respiratory viruses is one metapopulation. Studies should also focus on MERS-CoV exposure in humans with (e.g., herders, consumers) and without camel contact, together with an attempt to isolate and characterize the virus. Lastly, the absence of known human MERS-CoV cases in the Horn of Africa may be due to a lack of studies and thus warrants further investigation, including syndromic surveillance in humans.

## Supporting Information

S1 Fig Map of Laikipia County in central Kenya. (TIF)

S2 Fig Reactivity of sera from dromedary camels (*Camelus dromedarius*) in Laikipia County, Kenya with three coronavirus S1 antigens. (TIF)

S1 Table Seroprevalence to MERS-CoV Antibodies from nine herds of dromedary camels (*Camelus dromedarius*) in Laikipia County, Kenya. (DOCX)
