# Correction: Serological Evidence of MERS-CoV Antibodies in Dromedary Camels (*Camelus dromedaries*) in Laikipia County, Kenya

**DOI:** 10.1371/journal.pone.0178310

**Published:** 2017-05-18

**Authors:** The PLOS ONE Staff

The species name was spelled incorrectly in the article title. The correct title is: “Serological Evidence of MERS-CoV Antibodies in Dromedary Camels (*Camelus dromedarius*) in Laikipia County, Kenya”. The correct citation is: 1. Deem SL, Fèvre EM, Kinnaird M, Browne AS, Muloi D, Godeke G-J, et al. (2015) Serological Evidence of MERS-CoV Antibodies in Dromedary Camels (*Camelus dromedarius*) in Laikipia County, Kenya. PLoS ONE 10(10): e0140125. https://doi.org/10.1371/journal.pone.0140125

The publisher apologizes for the error.
