# Supplementary figures and images for: Serological Evidence of MERS-CoV Antibodies in Dromedary Camels (Camelus dromedaries) in Laikipia County, Kenya
Source: PLoS One. 2015 Oct 16;10(10):e0140125. doi: 10.1371/journal.pone.0140125 (PMC4608777; doi:10.1371/journal.pone.0140125)

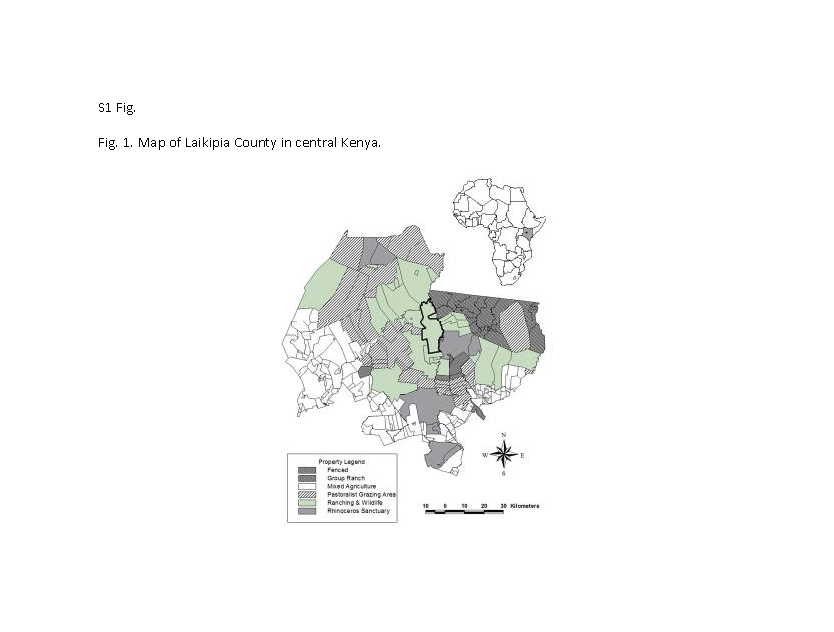

Supplement: S1 Fig — (TIF) [file pone.0140125.s001.tif]

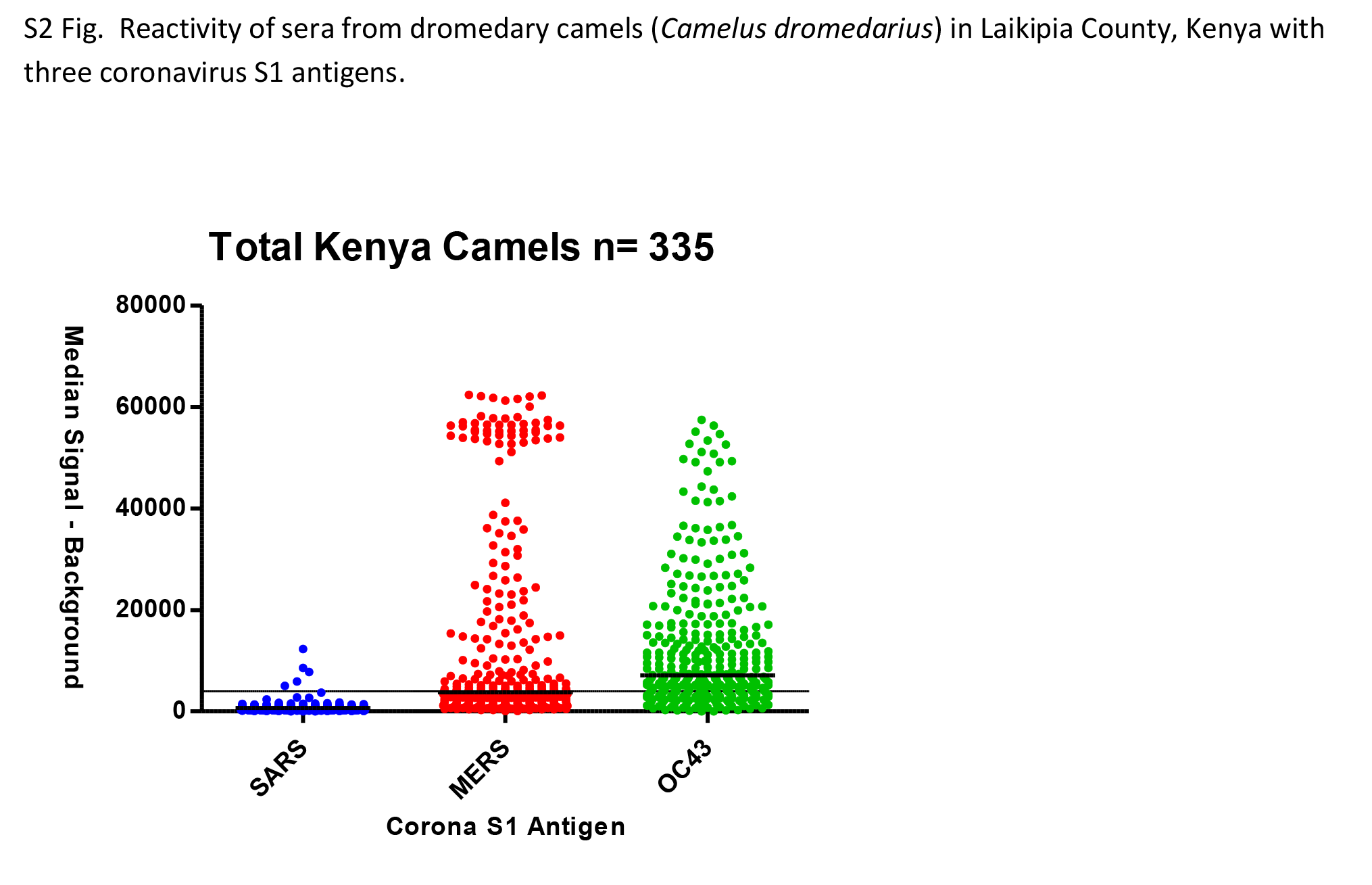

Supplement: S2 Fig — (TIF) [file pone.0140125.s002.tif]
