# Supplementary material for: Serological Evidence of MERS-CoV Antibodies in Dromedary Camels (Camelus dromedaries) in Laikipia County, Kenya
Source: PLoS One. 2015 Oct 16;10(10):e0140125. doi: 10.1371/journal.pone.0140125 (PMC4608777; doi:10.1371/journal.pone.0140125)
Supplement: S1 Table — (DOCX) [file pone.0140125.s003.docx]

S1 Table. Seroprevalence to MERS-CoV Antibodies from nine herds of dromedary camels (*Camelus dromedaries*) in Laikipia County, Kenya.

| **Ranch** | **Management Type** | **Herd Isolation** | **Herd Size** | **Adults** | **Juvenile** | **Young** | **All Ages** |
| --- | --- | --- | --- | --- | --- | --- | --- |
| A | Commercial | H | 47 | 23 / 24*  78.9 - 99.9** | 2 / 5  5.3 - 85.3 | 4 / 6  22.2 – 95.7 | 29 / 35  66.4 - 93.4 |
| B | Commercial | H | 50 | 8 / 30  12.3 - 45.9 | 4 / 15  7.8 - 55.1 | 1 / 5  0.51 – 71.6 | 13 / 50  14.6 - 40.3 |
| C | Commercial | H | 131 | 11 / 20  31.5 - 76.9 | 1 / 3  0.8 - 90.6 | 0 / 6  0 – 45.9 | 12 / 29  23.5 - 61.1 |
| D | Commercial | H | 257 | 3 / 19  3.4 - 39.6 | 10 / 10  69.2 - 100 | 0 / 6  0 – 45.9 | 5 / 35  4.8 - 30.3 |
| E | Commercial / Pastoralist | I | 18 | 10 / 13  46.5 - 90.3 | 0 / 3  0 – 70.8 | 1 / 2  1.3 – 98.7 | 11 / 18  35.7 - 82.7 |
| F | Commercial / Pastoralist | I | 76 | 14 / 18  52.4 - 93.6 | 0 / 10  0 – 30.8 | 2 / 6  4.3 – 77.7 | 16 / 34  29.8 - 64.9 |
| G | Nomadic | L | 34 | 18 / 26  48.2 - 85.7 | 1 / 1  2.5 – 100 | 0 / 0  0 – 0 | 19 / 27  49.8 - 86.2 |
| H | Nomadic | L | 122 | 16 / 24  44.7 - 84.4 | 4 / 13  9.1 – 61.4 | 3 / 10  6.7 – 65.2 | 23 / 47  34.1 - 63.9 |
| I | Commercial | L | 167 | 17 / 20  62.1 - 96.8 | 2 / 20  1.2 – 31.7 | 11 / 20  31.5 – 76.9 | 30 / 60  36.8 - 63.2 |
| Total |  |  | 902 | 118 / 194  53.6 - 67.7 | 17 / 80  12.9 – 31.8 | 24 / 61  27.1 – 52.7 | 157 / 335  41.4 - 52.5 |

| * number positive / number sampled  ** 95% lower confidence interval – upper confidence interval  Management Type: Commercial – for profit; commercial/pastoralist – group ownership;  nomadic – move across landscape for human and supply transport |
| --- |
|  |
|  |
| Herd Isolation: H = high (1 to 2 camels enter herd in 1 yr); I = intermediate (3-5 camels enter herd in 1 yr);  L = low (6 or more camels enter herd in 1 yr or camels move around consistently with high probability  of interacting with other camels)  Age: Adults > 2 years; juveniles, 6 months – 2 years; and young, ≤ 6 months |
